# Less overall, but more of the same: drivers of insect population trends lead to community homogenization

**DOI:** 10.1098/rsbl.2023.0007

**Published:** 2023-03-29

**Authors:** Martin M. Gossner, Florian Menzel, Nadja K. Simons

**Affiliations:** ^1^ Forest Entomology, Swiss Federal Research Institute WSL, Zürcherstrasse 111, Birmensdorf 8903, Switzerland; ^2^ Department of Environmental Systems Science, Institute of Terrestrial Ecosystems, ETH Zürich, Zürich 8092, Switzerland; ^3^ Institute of Organismic and Molecular Evolution, Faculty of Biology, Johannes Gutenberg University, Hanns-Dieter-Hüsch-Weg 15, Mainz 55128, Germany; ^4^ Ecological Networks, Department of Biology, Technical University of Darmstadt, Schnittspahnstraße 3, Darmstadt 64287, Germany

**Keywords:** insect decline, land-use change, climate change, invasive species, insect conservation, population trends

## 1. Introduction

The continuing decline in the diversity and biomass of insects and other arthropods has caused great concern not only among scientists, but also among society, policymakers and stakeholders. A major reason for this is that many ecosystem services depend on diverse insect communities. Despite numerous studies on the dynamics of insect communities [1,2], their causes are still not fully understood [3]. Rather than focusing on additional evidence of population declines, this special feature addresses the causes and consequences of population and diversity trends, aiming at a better mechanistic understanding of the observed dynamics.

**Figure 1.**
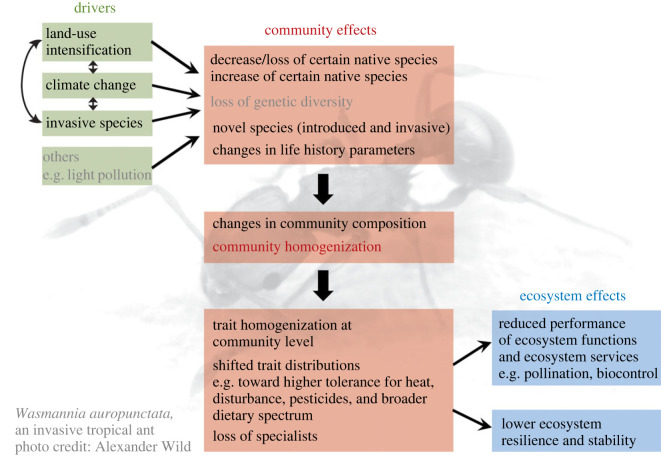
Overview of the relations between drivers of insect population trends and their effects on communities and ecosystems as described in this special feature. The aspects in grey are described in the literature but were not covered in this feature. The ant in the background is the invasive *Wasmannia auropunctata* (photo credit: Alexander Wild), which features in [4].

The special feature includes two opinion papers, 10 time-series analyses spanning 10 to 120 years and two studies using space-for-time substitution. The studies cover freshwater and terrestrial insect taxa across five biomes. The approaches are manifold, linking population trends to species-specific functional traits and examining spatial variation in population trends and their underlying drivers. Three of the major drivers of insect declines [1] are covered: climate change, land-use change and invasive species. Across the studies, one worrying pattern emerges: communities tend to become more homogeneous, i.e. lose beta diversity. This homogenization will likely have drastic consequences for ecosystem functioning and stability (figure 1).

## 2. Drivers of community change

### (a) Climate change

The warming climate influences both community composition and population dynamics of single species through changes in average or extreme temperatures. Among North American bumblebees, 37 of 46 studied species showed greater declines or lower increases in site occupancy under observed temperature changes than would have occurred if temperatures remained constant [5], suggesting that species have already reached their physiological limits in many regions. In addition, changes in precipitation patterns can alter population dynamics. For example, ant species that proliferated during the last decades in Denmark were associated with wet habitats, while declining ant species occurred in dry, open habitats [6]. In the same time span, average and frequency of precipitation had increased. The opposite effect was observed for two Orthoptera species in Germany, which severely decreased in wet and mesic grasslands over the study period (1988, 2004 and 2019), possibly due to summer droughts and increased evaporation [7].

As insects are ectotherms, their metabolism and development are driven by temperature, with warming typically resulting in faster development and higher metabolic rates [8,9]. However, extreme temperatures outside a species' optimum thermal range can slow development and thus reduce population growth rates [10,11]. This is especially relevant for tropical insects, which usually live closer to their upper thermal limit than their temperate counterparts [12,13]. Hence, global warming may be the main driver of tropical insect declines [4], favouring species that thrive under warmer conditions. Concerning species-specific climate-sensitivity traits, this was found in tiger moths in the field [14], but also in a warming experiment with ants [15], both in a Panamanian rainforest. In the temperate zone, similarly positive effects on thermophilous species were observed for orthopterans [7] and for stream-dwelling mayflies, stoneflies and caddisflies [16]. The shift to warm-adapted species thus appears as a more general global phenomenon confirmed by many other studies on individual species trends [17] and likely will result in an overall thermophilization of communities across many taxa [18,19]. In turn, cold-adapted species will migrate toward the poles or higher elevations [20,21], which can reduce their effective habitat area [22,23], thus increasing their extinction risk [24], ultimately accelerating biodiversity loss.

### (b) Land-use change

Land-use change and land-use intensification were identified decades ago as major causes of global biodiversity loss [25] and confirmed in several recent publications [2,26,27]. The papers in this special feature provide further evidence and highlight complex indirect effects that can cause insect declines. For example, as burning North American tallgrass prairie—traditionally used as a conservation measure—became less frequent over the last 34 years, grasshoppers needed more time for maturation [28]. This in turn contributed to declines in abundance as adults had less time to build egg mass before reproducing. Land-use changes in Denmark (1900–2019) challenged ant communities in several ways [6]. Three specialist species of dry, open habitats declined due to habitat decreases, attributed to conversion into agriculture and forest. In forest ecosystems, increased monocultures of coniferous plantations caused population declines in three species, while one species benefited from this change [6]. In German grasslands, fertilization contributed to species loss and an additive homogenization of grasshopper communities [7]. In Brazilian freshwater ecosystems, dam and hydroelectric power plant construction was pointed out as the main driver of abundance and richness declines in freshwater insects due to lower water turbidity and nitrogen increase [29]. Moreover, nutrient and pesticide inputs affected insect population dynamics in Swiss freshwater ecosystems [16]. All this underscores that land-use intensification is negatively impacting many species across taxa [1,26] resulting in homogenized communities [30,31] composed of species with distinctive traits that enable them to cope with increasing anthropogenic disturbances.

### (c) Invasive species

Biological invasions have increased massively in recent decades due to increased global trade and human movement [32,33] and are considered an important cause of biodiversity loss. Many invasive species negatively interact with or even displace native species [34], but the impacts on ecosystems can be complex and often indirect. In a Canadian forest, invasive earthworms directly and indirectly affect higher trophic levels mediated by plants, herbivores and detritivores [35]. Total arthropod abundance, biomass and species richness decreased significantly even at low levels of invasion. Another example comes from the subtropical freshwater ecosystems in Brazil, where the invasion of non-native insectivorous fishes appears as a major cause of freshwater insect declines over the last 20 years [29]. Overall, the impact of invasive species on ecosystems will probably keep increasing, which could particularly challenge species with low competitiveness.

### (d) Interactions among drivers

Drivers of insect decline may interact, such that combination effects on insect populations and communities can be more severe than the sum of single factors [36–38]. This special feature also provides evidence of such interactions. Interactions between land use and climate, and between land use and species invasions appear to be important drivers of declines across Brazilian biomes [39]. A decrease in vegetation cover through intensified land use, for instance, can reduce a habitat's potential to mitigate climate change-related drought (e.g. in an urban context [40]) or extreme temperatures (e.g. through deforestation, [41]). As another interaction, climate change can facilitate species invasions by favouring generalized, heat-tolerant species with invasive potential [15]. Often however, these interactive effects are hard to disentangle, which is why they are still poorly studied. For example, declines in freshwater insects were associated both with nutritional shifts in the water and with fish invasions [29], but it is hard to pinpoint the more important cause. In addition, other drivers of insect decline (e.g. light pollution [42]) have increased in impact over the past decades, making it even harder to disentangle such interactions.

## 3. Consequences for communities

Insect population trends are highly idiosyncratic, depending on taxonomic and functional groups. However, among the species *within* each group, certain traits were often associated with increasing or decreasing population trends. Winning species were usually warm-adapted or moderately heat-tolerant [7,14–16], tolerant to pesticides and disturbances [6,16], had invasive traits [15] and/or a broad dietary spectrum [16,43]. Decreasing species, in contrast, preferred dry, nitrogen-poor habitats [7] and open forests [6] or had a protein-rich diet [6]. This matches previous studies, which additionally identified high rates of dispersal and habitat recolonization after disturbance as traits associated with winners (e.g. [44]). Notably, climate change can select for different traits: depending on the region, species preferring wet conditions could be losers (Germany: [7]) or winners (Panama: [14]; Denmark: [6]). In addition, climate change could select for high migratory ability (i.e. dispersal rate) [24] and high thermal plasticity [45]. Genetically diverse species could also be at an advantage due to higher adaptability [46].

These trait changes combined with an increase of generalists likely increases the risk of homogenization. Thorn *et al.* [7] observed increasing homogenization of insect communities over time, i.e. a loss in alpha and beta diversity. Other studies find homogenizing effects on bumblebee and grasshopper communities [5,28]. Gebert *et al.* [16] argue that common taxa which are already less sensitive to extreme temperatures, become even more common in times of climate change, resulting in further homogenization. If generalist taxa also exhibit invasive traits (e.g. [15,16]), interspecific competition and species displacement becomes more likely especially as invasion rates are strongly accelerated both by global trade and climate change [47–50].

All these factors ultimately lead to ‘novel communities’ composed of introduced species and the surviving native ones [19]. New species may be beneficial for ecosystem functioning if they can substitute decreasing native species. However, a loss of species from the local or regional pool could result in lower functional redundancy and response diversity, thus reducing ecosystem stability and resilience to climatic variation or disturbance [51]. Besides, homogenization may directly lead to reduced functional performance, e.g. for interaction partners relying on specialists [30]. For example, a climate change-induced homogenization of alpine bumblebee communities led to a concomitant decline in plants specialized on long-tongued bumblebee pollinators [52,53].

## 4. Future directions

This special feature confirms that insect population trends vary a lot across taxa, regions and realms [39,54]. This may be because drivers differ in importance between regions. In addition, interconnections between realms or habitats make the effect of drivers context-dependent [55,56]. In one study, only 60% of co-occurring arthropod taxa at order level showed trends in the same direction [54]. Temporal trends in biomass, abundance and/or diversity are so variable that using only selected ‘bioindicator’ taxa, as commonly done in conservation, might not be sufficient to understand this variation and to develop effective conservation strategies. In addition, monitoring should consider abundances of species rather than those of entire taxonomic groups, as changes in community composition may go unnoticed if increases of one species mask decreases of others in the same group. Standardized ‘biodiversity monitoring stations' skilfully selected across biomes and realms with broad taxonomic and trophic coverage will be useful here.

Beside population trends, we should concomitantly monitor how they affect insect-mediated ecosystem functions such as pollination, decomposition, food for higher trophic levels and biocontrol. This way, we can also identify key species for particular functions [57] and understand how population dynamics will affect ecosystem functioning and stability alike. To identify vulnerable species and predict community changes, trait-based approaches will be useful, considering species-specific physiological traits (e.g. drought resistance, nutritional needs, ability to mature or diapause under changing climate) [58]. An important complement here is research on the plasticity and adaptive potential (e.g. genetic diversity) in different species [46,59]. In this context, we must keep in mind that abiotic and biotic conditions are dynamic and that the functional importance of a species may vary over time.

Despite the need for further research, there is already sufficient knowledge on how to mitigate species loss and promote biodiversity through political and individual actions [60–65]. The two opinion pieces in this special feature highlight the potential of approaches in addition to long-term monitoring. Weisser *et al*. [66] argue that we can already identify the most important drivers from quantitative analyses of already existing trend data, which should then be confirmed by driver-specific experiments. With an even shorter timeframe necessary, Blüthgen *et al.* argue that we can already conclude a lot from space-for-time approaches [67]. Both approaches provide scientific evidence for effective and targeted conservation or restoration measures. However, multiple approaches should be combined to avoid known issues inherent to each [3,68].

This special feature shows that there are complex interactions between major drivers of insect population dynamics and that effects vary between taxa, functional groups or ecosystems. Any implemented conservation measures should hence be accompanied scientifically to ensure their success [69,70]. But the main practical lesson from this is that we must manage habitats in a foresightful and adaptive way, anticipating unexpected developments. This may include habitat connectivity to allow the migration of species with climate change and enhancing local diversity to increase functional redundancy and thereby ecosystem stability. A network of well-selected protected areas designed for insect conservation, combined with integrative elements in managed landscapes can be valuable here [71]. Moreover, we need to put more effort into preventing and mitigating human-induced species invasions. Rather than only ‘more research’, we urgently need to realise conservation and habitat restoration measures known to effectively promote and protect insect populations and diverse communities to avoid further homogenization.

## Data Availability

This article has no additional data.
